# Rebound of Gonorrhea after Lifting of COVID-19 Preventive Measures, England

**DOI:** 10.3201/eid3002.231148

**Published:** 2024-02

**Authors:** Holly Fountain, Stephanie J. Migchelsen, Hannah Charles, Tika Ram, Helen Fifer, Hamish Mohammed, Katy Sinka

**Affiliations:** UK Health Security Agency, London, UK

**Keywords:** gonorrhea, COVID-19 restrictions, COVID-19 preventive measures, COVID-19, respiratory infections, severe acute respiratory syndrome coronavirus 2, SARS-CoV-2, SARS, coronavirus disease, zoonoses, viruses, bacteria, Neisseria gonorrhoeae, coronavirus, England, United Kingdom

## Abstract

After lifting of all COVID-19 preventive measures in England in July 2021, marked, widespread increases in gonorrhea diagnoses, but not testing numbers, were observed, particularly in persons 15–24 years of age. Continued close surveillance and public health messaging to young persons are needed to control and prevent gonorrhea transmission.

The COVID-19 pandemic caused a substantial disruption of sexual health services (SHS) in England (including reduced testing), contributing to a 33.5% decrease in new sexually transmitted infection (STI) diagnoses in 2020 (n = 311,480) compared with 2019 (n = 468,260) (*1*). In July 2021, all COVID-19 restrictions associated with the third and final lockdown in England were lifted (*2*), normal social mixing was permitted, and a rebound in SHS occurred; a 23.8% increase in new STI diagnoses was observed in 2022 (n = 392,453) compared with those in 2021 (n = 317,022) (*1*). Of the most commonly diagnosed STIs, the largest proportional increase occurred for gonorrhea, caused by infection with *Neisseria gonorrhoeae* bacteria. The number of new gonorrhea diagnoses increased by 50.3% in 2022 (n = 82,592) compared with 2021 (n = 54,961) (*1*). We describe trends for gonorrhea testing and diagnosis in England after all COVID-19 control measures were lifted and explore how those differed among populations.

## The Study

In England, all STI tests and diagnoses from SHS are captured by the Genitourinary Medicine Clinic Activity Dataset STI Surveillance System (*3*). We analyzed data on gonorrhea tests and diagnoses during January 1, 2019–December 31, 2022. To prevent double counting, we only counted 1 test or diagnosis per SHS user within a 42-day period. We examined quarterly trends, disaggregated by age group (15–24, 25–34, 35–44, and >45 years of age), gender and sexual orientation (gay, bisexual, and other men who have sex with men [MSM]; heterosexual men; women who have sex with men [WSM]; and women who only have sex with women), and local authority districts of residence. We did not include records with missing demographic data in their respective analysis. We analyzed data by using Stata version 16.1 software (StataCorp LLC, https://www.stata.com). No ethics approval was needed for this study because we used routine surveillance data.

**Figure 1:**
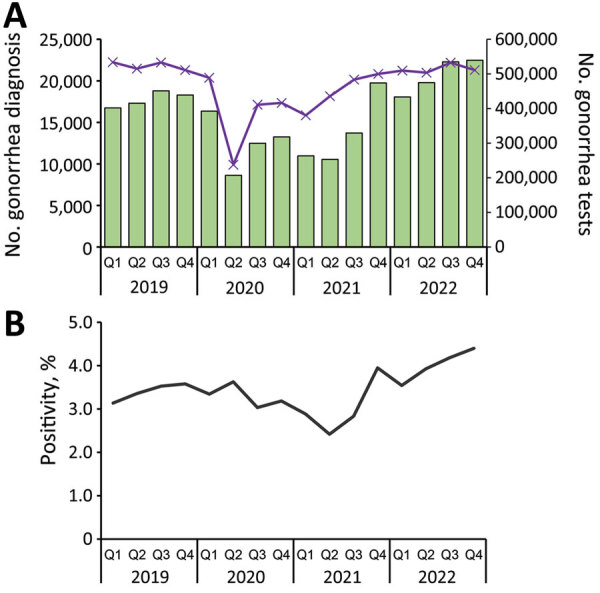
Total number of gonorrhea diagnoses and tests and percent test positivity in study of rebound of gonorrhea after lifting of COVID-19 preventive measures in England, January 1, 2019–December 31, 2022. A) Total number of diagnoses and tests. Bars indicate the total number of gonorrhea diagnoses; purple line indicates the total number of gonorrhea tests. B) Percent positivity of gonorrhea tests. Q, quarter. Scales for the y-axes differ substantially to underscore patterns but do not permit direct comparisons.

After lifting of COVID-19–related restrictions, the number of gonorrhea tests increased by 5.6% from quarter (Q) 3 of 2021 (n = 483,717) to the end of Q4 of 2022 (n = 510,792). Gonorrhea diagnoses increased by 63.8% (13,715 to 22,471) during the same period (Figure 1, panel A). The total number of gonorrhea diagnoses in 2022 was the highest on record, although testing remained just below 2019 levels. Test positivity increased to the highest point within the 4-year study period during 2022 Q4 (4.4%) from a low point in 2021 Q3 (2.8%) (Figure 1, panel B).

**Figure 2:**
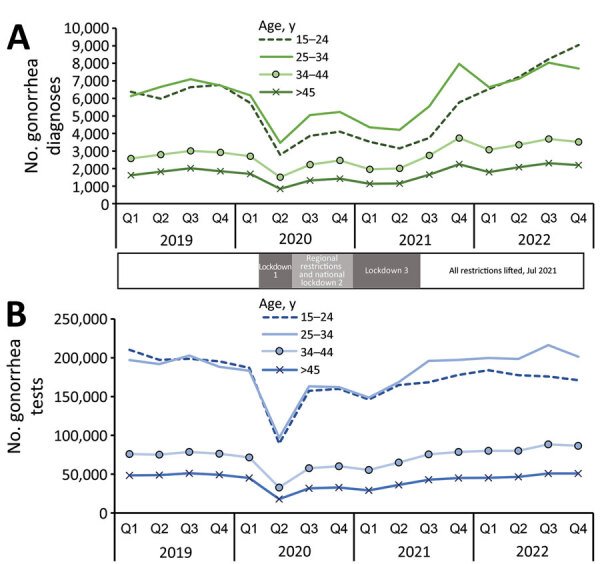
Total number of gonorrhea diagnoses (A) and tests (B) according to age groups in study of rebound of gonorrhea after lifting of COVID-19 preventive measures in England, January 1, 2019–December 31, 2022. Shaded bars between panels indicate dates of COVID-19 lockdowns. Q, quarter.

Increases in gonorrhea diagnoses began immediately after COVID-19–related restrictions were lifted and were most notable in young persons, 15–24 years of age, who saw a 141.3% increase (3,747 diagnoses in 2021 Q3, 9,041 in 2022 Q4) (Figure 2, panel A); a 34.8% increase was observed for persons >25 years of age. Persons 19–20 years of age had the highest increase in diagnoses, 229.0% (930 in 2021 Q3, 3,060 in 2022 Q4). During the same period, testing remained relatively steady among young persons (1.5% increase). Overall, testing returned to or exceeded numbers from 2019 in all age groups except the 15–24-year group (12.4% decrease from 2019 Q4 to 2022 Q4) (Figure 2, panel B). Test positivity increased >2-fold in persons 15–24 years of age from 2021 Q3 (2.2%) to 2022 Q4 (5.3%); positivity increased at a lower rate in older age groups.

Of all gender and sexual orientation groups, MSM had the largest numbers of gonorrhea diagnoses during the 4-year study period. However, proportionally, increases in gonorrhea diagnoses from 2021 Q3 to 2022 Q4 were largest among WSM (104.7% increase; 2,577 to 5,274) and heterosexual men (90.4%; 2,152 to 4,097); diagnoses among MSM increased 39.7% (7,107 to 9,932) (Table 1). Testing increased by 21.8% among MSM from 2019 Q4 to 2022 Q4; however, testing decreased by 20.3% for WSM and by 8.1% for heterosexual men during the same period. Among persons 15–24 years of age, the increase in gonorrhea diagnoses from 2021 Q3 to 2022 Q4 was greater among heterosexual persons (179.6%; 2,092 to 5,850) than among MSM (63.2%; 1,035 to 1,689). For persons >25 years of age, diagnoses increased similarly among heterosexual persons (33.7%) and MSM (35.8%).

**Table 1:** Numbers of persons with gonorrhea diagnoses who attended sexual health services according to gender and sexual orientation in study of rebound of gonorrhea after lifting of COVID-19 preventive measures, England*

Year	No. persons with gonorrhea diagnosis				
	MSM	Heterosexual men	WSM	WOSW	Not known
2019					
Quarter 1	7,851	3,678	4,280	44	889
Quarter 2	8,341	3,692	4,205	64	1,005
Quarter 3	9,249	3,832	4,498	55	1,152
Quarter 4	8,466	3,909	4,757	47	1,119
2020					
Quarter 1	8,040	3,356	3,883	60	1,005
Quarter 2	4,352	1,681	1,962	39	580
Quarter 3	6,194	2,436	2,832	59	956
Quarter 4	6,491	2,585	2,964	69	1,134
2021					
Quarter 1	5,184	2,041	2,392	52	1,305
Quarter 2	5,251	1,674	2,076	77	1,456
Quarter 3	7,107	2,152	2,577	69	1,810
Quarter 4	8,724	2,674	3,665	85	4,590
2022					
Quarter 1	8,809	2,916	3,809	109	2,419
Quarter 2	9,684	3,224	4,182	127	2,560
Quarter 3	10,498	3,684	5,032	115	2,953
Quarter 4	9,932	4,097	5,274	123	3,045

*Preventive measures were lifted in July (quarter 3) of 2021. MSM, gay, bisexual and other men who have sex with men; WOSW, women who only have sex with women; WSM, women who have sex with men.

Gonorrhea diagnoses increased in all regions of England after COVID-19–related restrictions were removed, most notably in South West England (226.0% increase; 407 in 2021 Q3 to 1,327 in 2022 Q4) and North East England (194.0% increase; 285 in 2021 Q3 to 838 in 2022 Q4). Most (91.3%) local authority districts showed an increase in diagnoses during this same period.

## Conclusions

National surveillance data showed an increase in gonorrhea diagnoses in England after the cessation of social restrictions in summer 2021. Increases were observed among persons of all age groups, genders, and sexual orientations but particularly among persons 15–24 years of age and those who identified as heterosexual.

Testing did not increase as markedly as diagnoses after the removal of COVID-19 lockdown restrictions, suggesting that a true increase in gonorrhea transmission existed within the population. Changes in diagnosis numbers could potentially represent a delay in gonorrhea detection because of decreased testing during the lockdown periods; however, this difference is unlikely to be a main factor for the observed increase in diagnoses because the largest increases in testing primarily occurred before all lockdown restrictions were removed (i.e., from 2020 Q2 to Q3 and 2021 Q1 to Q3; Figure 1, panel A). Furthermore, 18 months after lockdowns ended, increases in gonorrhea diagnoses continued to outpace increases in testing, corresponding to increasing test positivity. In addition, gonorrhea in heterosexual men is likely to cause symptomatic urethritis, which might cause those persons to seek earlier treatment at SHS. Although we did not have access to data on symptoms for confirmation, the increase in diagnoses observed in heterosexual men suggests an increase in incident infection and not just a delay in detection.

The sexual behavior of young persons was most adversely affected by COVID-19–related restrictions (*4*). After all lockdown restrictions were removed during summer 2021, the return of in-person attendance at higher education institutions might have provided increased opportunities for new and frequent changing of sexual partners. The potential change to higher STI risk behaviors could perhaps help explain the observed increases and is similar to reports from other countries in Europe in which gonorrhea diagnoses increased in 2022 among young persons of average university age (*5*–*8*).

Young persons were affected the most by disruptions to SHS caused by lockdown measures (*9*). Testing by using online services increased, but evidence of access inequality existed; those 15–19 years of age were less likely to access testing through this route (*10*). We have shown that testing levels in young persons remained lower in 2022 than during the prepandemic period, suggesting that potential undiagnosed cases contributed to observed increases in gonorrhea. Messaging focused on the importance of regular STI testing, especially for young persons with new partners, could help address disease transmission resulting from undiagnosed cases. Public health messaging directed at young persons was published to coincide with the start of the 2023–24 academic year (*11*).

In conclusion, the increase in gonorrhea diagnoses was widespread in England after removal of all COVID-19 lockdown restrictions. It remains to be seen whether increases in gonorrhea diagnoses will be short-lived because of restrictions removal, whether pre–COVID-19 pandemic diagnoses levels will resume, or whether another trend will be observed. Continued close surveillance, a better understanding of the factors leading to the increase in gonorrhea diagnoses, and public health messaging (particularly to young persons) are needed to focus efforts on gonorrhea transmission control and prevention.
